# Associations Between Physical Fitness and Health-Related Quality of Life in Children with Obesity

**DOI:** 10.3390/diagnostics16030371

**Published:** 2026-01-23

**Authors:** Aldona Wierzbicka-Rucińska, Anna Wrona, Mieczysław Szalecki, Joanna Mazur, Jacek Podogrodzki

**Affiliations:** 1 Department of Clinical Biochemistry, The Children’s Memorial Health Institute, Aleja Dzieci Polskich 20, 04-730 Warsaw, Poland; 2 Department of Science, The Children’s Memorial Health Institute, Aleja Dzieci Polskich 20, 04-730 Warsaw, Poland; 3 Clinic of Endocrinology and Diabetology, The Children’s Memorial Health Institute, Aleja Dzieci Polskich 20, 04-730 Warsaw, Poland; 4 Department of Humanization of Medicine and Sexology, Collegium Medicum, University of Zielona Gora, 65-046 Zielona Gora, Poland; 5 Department of Neurology and Epileptology, The Children’s Memorial Health Institute, Aleja Dzieci Polskich 20, 04-730 Warsaw, Poland

**Keywords:** quality of life, health-related quality of life (HRQoL), physical fitness, simple obesity, childhood obesity, adolescents, EUROFIT test, psychosocial factors

## Abstract

Obesity is associated with multiple comorbidities and therefore requires a multidisciplinary approach. Particular attention is given to the role of visceral adiposity and its impact on quality of life. Childhood obesity, in particular, is a major global public health challenge with physical, psychological, and social consequences extending into adulthood. Within the framework of personalized medicine, assessing physical fitness and health-related quality of life (HRQoL) offers valuable insight for defining individualized therapeutic goals. **Objective:** To evaluate the relationship between physical fitness and HRQoL in children with simple obesity and to highlight the potential value of personalized approaches in pediatric obesity management. **Methods:** This study included 123 patients aged 8–16 years with simple obesity who were hospitalized at the Children’s Memorial Health Institute in Warsaw. Obesity was diagnosed according to CDC growth charts (OLAF study). Physical fitness was assessed using the EUROFIT test battery (8 trials), and HRQoL was measured with the Kidscreen-52 questionnaire (10 domains). **Results:** The overall EUROFIT test performance in the study group was significantly lower compared with population norms (*p* < 0.001). Similarly, HRQoL scores reported by both children and their parents were significantly below reference values (*p* < 0.001). **Conclusions:** Reduced physical fitness is strongly associated with impaired quality of life in children with obesity. Personalized interventions aimed at improving motor performance may represent an effective strategy in the prevention and treatment of pediatric obesity.

## 1. Introduction

Obesity in children and adolescents is a multifactorial disease influenced by biological, behavioral, psychosocial, and environmental factors. Morbid obesity, defined by a body mass index (BMI) ≥ 40 kg/m^2^ or ≥35 kg/m^2^ with obesity-related comorbidities, presents complex diagnostic challenges that demand a multidisciplinary approach. This condition requires the integration of anthropometric, metabolic, imaging, and biochemical markers to improve phenotyping and risk stratification. Key considerations include the limitations of BMI as a sole diagnostic tool, the importance of visceral adiposity imaging, and the emerging role of genetic and epigenetic profiling.

Moreover, comorbidities such as metabolic syndrome and obstructive sleep apnea substantially modify clinical trajectories and therapeutic outcomes. By bridging gaps between clinical evaluation and advanced diagnostic methods, this framework aims to enhance early identification and improve management strategies for high-risk populations. Evidence shows that children with obesity often avoid or limit physical activity [1]. According to the World Health Organization, physical fitness is defined as the ability to perform effective muscular work [2]. Wasiluk and Saczuk describe health-related fitness (HRF) as a state of positive health associated with global and local socioeconomic factors [3]. Similarly, Bouchard and Shepard highlight that HRF refers to components of fitness that directly influence health, including cardiorespiratory endurance, daily activity levels, and thermoregulation [4]. Quality of life is a multidisciplinary construct encompassing psychology, sociology, economics, pedagogy, and medicine [5,6]. Its health-related variant (HRQoL) is typically categorized into four domains: physical and motor fitness, mental well-being, social and material conditions, and somatic health perceptions. Taken together, these definitions suggest a strong interdependence between physical fitness and HRQoL. Evaluating both domains in children with obesity may therefore provide an important foundation for understanding health outcomes [7]. Measuring health-related quality of life is currently a key method of self-assessing health, enabling the detection of disturbances in the biopsychosocial functioning of children and adolescents. The World Health Organization (WHO) indicates that quality of life should be assessed across four dimensions: physical health, psychological health, social health, and environmental relationships [8]. The term Health-Related Quality of Life (HRQoL) is based on a multidimensional approach to the concept of health [9]. Detailed definitions focus on dimensions encompassing subjective assessments of one’s own health status [10]. The eating behaviors of children and adolescents are a key element of a healthy lifestyle.

The purpose of this study was to investigate the relationship between physical fitness, assessed with the EUROFIT test, and health-related quality of life (HRQoL), measured using the KIDSCREEN-52 questionnaire, in children and adolescents with simple obesity, as well as to identify factors influencing health outcomes in this population.

## 2. Materials and Methods

Study Design and Participants: This cross-sectional study included 123 children and adolescents (64 boys, 52%; 59 girls, 48%) aged 8–16 years who were treated at the Endocrinology and Diabetology Clinic and Pediatric Rehabilitation Center of The Children's Memorial Health Institute (IP-CZD), Warsaw, Poland. All participants were diagnosed with simple obesity according to CDC percentile charts based on the OLAF study. Written informed consent was obtained from parents or legal guardians, and the study protocol was approved by the institutional ethics committee.

The actual sample size was determined based on the availability of patients meeting the inclusion criteria during the study period. A total of 123 children and adolescents aged 8–16 years, hospitalized at The Children’s Memorial Health Institute in Warsaw, with simple obesity diagnosed according to CDC growth charts (OLAF study), were included in the analysis. The sample size was considered sufficient to obtain statistically significant results in the analysis of the relationship between physical fitness and health-related quality of life (HRQoL).

**Inclusion criteria:** Age 8–16 years, diagnosis of simple obesity (BMI ≥ 95th percentile according to CDC/OLAF growth charts), written consent from parents/legal guardians to participate in the study. Absence of chronic diseases or genetic disorders that could affect physical fitness or HRQoL outcomes.

**Exclusion criteria:** Presence of chronic diseases or metabolic disorders other than simple obesity. Conditions limiting physical activity (e.g., musculoskeletal disorders, cardiovascular diseases). Lack of consent from parents/legal guardians or the child to participate in the study.

### 2.1. Physical Fitness Assessment

Physical fitness was assessed using the EUROFIT test battery, which comprises eight trials evaluating different components of motor performance, including flexibility, muscular strength, endurance, and coordination. The results were compared with normative population data to determine deviations in physical fitness levels among the study participants. The battery includes the following tests: Flamingo Balance Test (static balance), Plate Tapping (upper-limb speed and coordination), Sit-and-Reach (flexibility), Standing Broad Jump (explosive leg power), Handgrip Strength (maximal isometric strength), Sit-Ups in 30 s (abdominal muscular endurance), Bent Arm Hang (upper-body muscular endurance), 10 × 5 m Shuttle Run (speed and agility), and the 20 m Endurance Shuttle Run (cardiorespiratory endurance). All tests were conducted according to the EUROFIT manual under standardized conditions by trained examiners. In this study, eight of the nine tests were used. The endurance component (20 m shuttle run) was excluded due to patient safety concerns and the inability to perform the running test in a hospital environment. Test results were expressed on a scale from 0 to 100 points. The scores were referenced against normative population data published by the Academy of Physical Education (AWF) in Poland.

### 2.2. Health-Related Quality of Life Assessment

Health-related quality of life (HRQoL) was measured using the Kidscreen-52 questionnaire, a validated self-assessment tool for children and adolescents. This 52-item instrument evaluates 10 dimensions of HRQoL: physical well-being, psychological well-being, moods and emotions, self-esteem, autonomy, parent relations and home life, social support and peers, school environment, social acceptance (including bullying), and financial resources. Sample items include the following: “In general, how would you rate your health?” (physical well-being), “Have you felt satisfied with your life?” (moods and emotions), and “Are you satisfied with the way you are?” (self-esteem). Cronbach’s alpha for the questionnaire dimensions ranged from 0.77 to 0.89, indicating good internal consistency. Higher scores generally reflect better HRQoL, except for the mood and bullying dimensions.

W1 Physical Well-being
W2 Psychological Well-being
W3 Moods and Emotions
W4 Self-Perception
W5 Autonomy
W6 Parent Relation and Home Life
W7 Social Support and Peers
W8 School Environment
W9 Social Acceptance and Bullying
W10 Financial Resources

### 2.3. Physical Activity Assessment

Participants’ physical activity levels were evaluated using the Physical Activity Questionnaire for Children and Adolescents, which captures frequency and intensity of daily activity in school and leisure settings. This provided complementary data to examine the relationship between activity levels, physical fitness, and HRQoL.

### 2.4. Statistical Analysis

Descriptive statistics were calculated for all variables. Continuous variables are presented as mean ± standard deviation (SD), while categorical variables are presented as frequencies and percentages. Comparisons between study participants and normative population data were performed using one-sample *t*-tests or chi-square tests, as appropriate. Correlations between physical fitness components and HRQoL dimensions were analyzed using Pearson’s or Spearman’s correlation coefficients depending on data distribution. Statistical significance was set at *p* < 0.05. All analyses were conducted using SPSS version 25.

## 3. Results

### 3.1. Physical Fitness

The overall EUROFIT scores were significantly lower than population norms for the total group (*N* = 123, *p* < 0.00001), as well as for girls (*p* < 0.00001) and boys (*p* < 0.00001) analyzed separately. Specifically, balance, upper-limb movement speed, jumping ability, trunk strength, functional strength, and agility were significantly below normative values (all *p* < 0.00001). Flexibility remained within the normal range (*p* = 0.38), while hand strength exceeded the normative values (*p* < 0.00001). Additionally, girls performed significantly better than boys in flexibility, jumping, hand strength, and agility. These results are presented in Table 1.

### 3.2. Health-Related Quality of Life

The quality-of-life results in the entire study group, in the opinion of children (*N* = 123, *p* = 0.00001) and their parents (*N* = 123, *p* = 0.00001), were significantly lower than the norm; among boys (*N* = 64, *p* = 0.07) and their parents (*N* = 64, *p* = 0.08), they showed a tendency to values lower than the norm, and among girls (*N* = 59, *p* = 0.00001) and their parents (*N* = 59, *p* = 0.00001), they were significantly lower. These results are presented in Table 2.

In Table 3, significant positive correlations were found in girls from the study group between balance and W10; upper limb movement speed and W4; flexibility and W3 and W4; hand strength and W1 and W8; as well as significant negative correlations between trunk strength and W6 and W9.

In boys from the study group, significant positive correlations were found between hand strength and W3, W6, and W9, as well as between trunk strength and W3 (Table 4).

Significant correlations between standardized HRQoL scores assessed by parents of girls from the study group and motor test results are presented in Table 5. Positive correlations were found between W3, W4 and flexibility, as well as between W5, W8 and hand strength. Negative correlations were observed between W6, W9 and trunk strength; between W6 and functional strength; and between W9 and agility.

Table 6 shows significant positive correlations between standardized HRQoL scores assessed by parents of boys from the study group and motor test results: flexibility was positively correlated with W3, W4, and W8; hand strength with W2, W3, W4, and W5; and trunk strength with W4.

**Figure 1:**
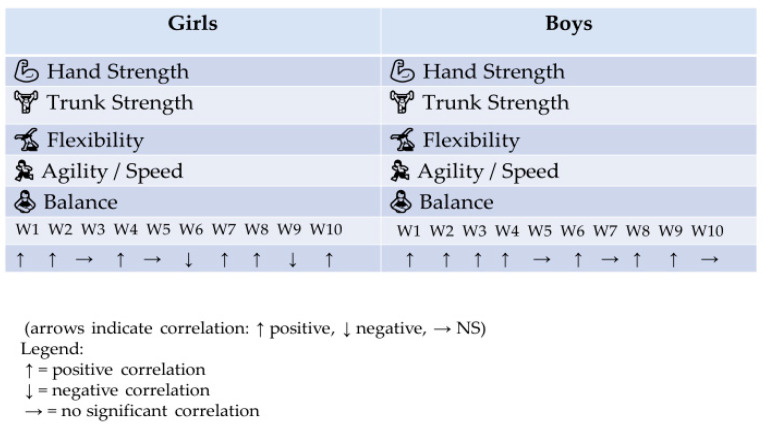
Correlation between quality of life and physical fitness in the study group.

Both girls and boys show strong positive associations between trunk and hand strength and the measured factors. Girls tend to show more variability in flexibility correlations, while boys’ correlations are more concentrated in strength and agility. Some factors show no significant correlation with certain components, indicating that not all aspects of fitness are equally influenced by these factors (Figure 1).

## 4. Discussion

This study focused on two key aspects of children’s and adolescents’ lives: the physical dimension, which is fundamental for optimal use of the body, and the psychosocial dimension, which reflects life satisfaction and relationships with the environment [11]. Our analyses demonstrated that these areas are closely interconnected. Motor test results revealed a marked deficit in physical fitness among children and adolescents with obesity (Table 1). Likewise, HRQoL scores indicated a significant decline compared with population norms (Table 2). Reduced physical fitness was associated with impaired health, psychological well-being, and social functioning, limiting opportunities for social integration and future professional development. Children with obesity often depend more heavily on healthcare and family support, while their relationships at home and at school may also be negatively affected [12]. Among the eight motor skills assessed, hand strength and flexibility emerged as particularly important in relation to HRQoL. Across the whole study group, hand strength scores were significantly above the population norm, and flexibility remained within the norm. Both correlated with multiple HRQoL domains—six in total (Table 3 and Table 4). In girls, flexibility was positively associated with moods and emotions as well as self-perception, highlighting its beneficial impact on mental health and self-image. Girls performed significantly better than boys in flexibility, which may partly explain these findings. Hand strength also correlated positively with indices of physical health and social support. Strength is widely recognized as a marker of health and is often valued by peers. In parental assessments, hand strength was positively related not only to social support but also to autonomy, suggesting that parents may perceive strength as fostering greater independence. Additional associations were observed between balance and social acceptance, as well as between upper limb movement speed and self-perception in girls. These findings indicate that motor coordination supports peer acceptance, while speed contributes to efficiency in daily activities and a more positive self-concept. Conversely, negative correlations between trunk strength and certain HRQoL domains (home life, school environment) in girls may be explained by sexual dimorphism, as this trait could be perceived negatively when associated with greater waist circumference. In boys, hand strength was strongly and consistently linked with HRQoL, correlating positively with moods and emotions, relationships with parents, home life and the school environment. Parental reports also highlighted associations with mental well-being, self-perception, and home life. These findings underscore the critical role of strength in shaping boys’ psychological well-being and social functioning. Flexibility was also associated with moods, emotions, and self-perception, although the effect was weaker than for other motor skills. Our findings are consistent with international studies. Morales et al. [8] examined 1158 Spanish simple school students and found significant positive correlations between cardiorespiratory fitness and HRQoL domains, particularly physical health, social support, self-perception, and social acceptance [13,14]. Similarly, our results demonstrated that hand strength and flexibility were strongly related to physical health and social domains, particularly in girls. Children with obesity exhibited lower physical activity and poorer HRQoL compared with their normal-weight peers [15,16]. Obesity and obese Spanish children had significantly lower physical fitness and HRQoL than controls, except for hand strength and certain social domains in boys [17,18]. Importantly, their mediation analyses indicated that physical fitness moderated the negative impact of obesity on HRQoL [19], which aligns with our observation that better fitness (particularly strength and flexibility) mitigates impairments in quality of life. The positive effects of physical fitness on selected HRQoL domains demonstrated in previous studies are confirmed by our findings. Hand strength and flexibility—both relatively preserved in our cohort—were distinguished by the highest number of significant positive associations with quality of life domains (17 across children and parents). These results highlight the potential of motor skill development as a target for personalized interventions in pediatric obesity. The COVID-19 pandemic has further underscored the importance of promoting physical activity [20,21]. WHO recommends 150–300 min of moderate-intensity or 75–150 min of vigorous-intensity physical activity per week for children and adolescents. However, sedentary behaviors such as prolonged screen time have been linked to an increased risk of depression and cardiovascular disease [22]. Identifying children with obesity who are particularly vulnerable to sedentary lifestyles is therefore essential for early preventive strategies.

Simple obesity in children and adolescents is associated with a pronounced reduction in physical fitness, particularly in tests that require moving body mass against gravity, such as standing broad jumps, agility, and trunk strength. Flexibility was the least affected component, with results generally comparable to population norms, especially among girls, which may reflect sex-related biological predispositions. Fat mass and waist circumference showed the strongest negative correlations with fitness scores, confirming their role as key limiting factors in dynamic and endurance-based performance. The EUROFIT test battery proves to be a valuable tool for assessing physical fitness in children and adolescents with obesity or obesity, enabling identification of the specific motor abilities most impacted by excess body weight [23].

### 4.1. Practical Application

For girls, it is advisable to introduce more hand and trunk strength exercises, as they improve physical well-being, school functioning, and social relationships. Emphasis on flexibility and coordination exercises strongly influences self-esteem, mood, and perceived attractiveness. Building a supportive peer environment strengthens both the physical and psychological development of girls. It is important to pay attention to girls who are exceptionally strong physically, as they may experience difficulties at home or in relationships. Ensuring equal access to sports activities is also crucial, because material resources correlate with fitness outcomes. For boys, strength is a key factor in well-being, as it affects emotions, self-esteem, family relationships, and social acceptance. Building strength improves both mental and social health—functional and strength training serve as therapeutic tools. Agility and coordination support peer relationships and school adaptation. Unlike in girls, there are no negative correlations with physical fitness; it is a “safe” factor for boys’ well-being. Physical activity programs can serve a preventive role (anti-depressive and anti-bullying).

### 4.2. Strengths and Limitations

Strengths of this study include the focus on children and adolescents with simple obesity without other medical conditions, allowing clearer interpretation of the associations between fitness, HRQoL, and body composition. Additionally, the combined evaluation of motor performance and HRQoL provides a foundation for personalized recommendations in obesity management. Limitations include the absence of dietary data, which could have enriched the analysis of lifestyle factors, and the cross-sectional design, which precludes causal inferences. Future studies should incorporate more detailed anthropometric and body composition measurements, as well as dietary and longitudinal data, to improve the understanding of how physical fitness interacts with HRQoL in this population.

## 5. Conclusions

This study demonstrates a significant association between body weight categories and selected dimensions of health-related quality of life (HRQoL) in adolescents. Obesity was particularly associated with reduced psychological well-being, lower self-esteem, and increased exposure to bullying, irrespective of gender. These findings highlight the necessity for weight management strategies that address not only metabolic and physiological outcomes but also psychological and social aspects. Effective management of morbid obesity requires a multidisciplinary approach that integrates physical fitness, lifestyle factors, and psychosocial components to mitigate the broader health consequences of excess body weight. Such comprehensive strategies may improve psychological well-being, enhance self-perception, and reduce vulnerability to adverse social outcomes. Future public health policies should incorporate lifestyle, metabolic, and quality of life indicators to support accurate diagnosis and targeted interventions. The implementation of assessment tools covering nutritional status, physical activity, and psychosocial determinants will aid clinicians in optimizing management plans, ultimately improving health outcomes in adolescents affected by morbid obesity. Effective management of morbid obesity requires a multidisciplinary approach that integrates physical fitness, lifestyle factors, and psychosocial components to mitigate the broader health consequences of excess body weight. Such comprehensive strategies may improve psychological well-being, enhance self-perception, and reduce vulnerability to adverse social outcomes. Future public health policies should incorporate lifestyle, metabolic, and quality-of-life indicators to support accurate diagnosis and targeted interventions. The implementation of assessment tools covering nutritional status, physical activity, and psychosocial determinants will aid clinicians in optimizing management plans, ultimately improving health outcomes in adolescents affected by morbid obesity. In the study group of children and adolescents with simple obesity, a significant reduction in quality of life measured with the KIDSCREEN-52 questionnaire was found in both the children’s self-assessments and their parents’ evaluations across most domains. This reduction was particularly pronounced in the physical health (W-1) and self-perception (W-4) indices, which were at levels comparable to those observed in severe chronic diseases. Collectively, the results reveal that although most correlations between physical performance and HRQoL were weak and non-significant, selected physical well-being—particularly flexibility, hand strength, trunk strength, and agility—showed meaningful associations with specific dimensions of psychosocial well-being.

## Figures and Tables

**Table 1:** EUROFIT test results in the study group.

	Gender, *N*	Mean	Min	Max	SD	Norm	*p*-Value
Average sum of 8 performance attributes (points)	Total *N* = 123	33.48	12.50	46.50	±6.70	50	*p* < 0.05
	Girls, *N* = 59	34.95	23.13	46.25	±5.30	50	*p* < 0.05
	Boys, *N* = 64	32.13	12.50	46.50	±7.56	50	*p* < 0.05

Statistically significant difference vs. population norm, *p* < 0.05.

**Table 2:** Summary scores (Z-scores) of HRQoL dimensions in the study group.

Group	Mean	Min	Max	SD	Norm	*p*-Value
Children (total)	−0.39	−2.5	2.19	0.83	0.00	<0.05
Parents (total)	−0.36	−2.09	2.37	0.75	0.00	<0.05
Boys (children)	−0.20	−2.57	2.19	0.88	0.00	NS
Girls (children)	−0.59	−2.22	1.45	0.72	0.00	<0.05
Boys (parents)	−0.17	−2.02	2.37	0.77	0.00	NS
Girls (parents)	−0.56	−2.09	1.12	0.67	0.00	<0.05

NS: not significant. Statistically significant difference, *p* < 0.05.

**Table 3:** Pearson’s r linear correlations between the results of the fitness test and the standardized (Z-score) results of the self-assessment of the quality of life of girls from the study group.

*N* = 59 Girls	W1 Z-Score	W2 Z-Score	W3 Z-Score	W4 Z-Score	W5 Z-Score	W6 Z-Score	W7 Z-Score	W8 Z-Score	W9 Z-Score	W10 Z-Score
Balance (points-pts)	0.02	−0.01	−0.07	0.12	0.00	−0.06	0.01	0.00	0.04	0.27 *
Upper Limb Movement Speed (pts)	0.17	0.13	0.16	0.30 *	0.10	−0.07	0.16	0.17	0.01	0.07
Flexibility (pts)	0.13	0.11	0.29 *	0.26 *	−0.05	−0.14	0.11	0.15	0.11	0.16
Jumping Ability (pts)	0.06	−0.04	−0.03	0.24	−0.01	−0.24	0.02	−0.10	−0.22	0.10
Hand Strength (pts)	0.28 *	0.10	−0.18	0.08	0.19	0.03	0.23	0.37 *	0.15	0.05
Trunk Strength (pts)	0.05	−0.19	0.02	0.16	−0.13	−0.30 *	−0.05	−0.04	−0.26 *	0.18
Functional Strength (pts)	0.14	−0.23	−0.23	−0.21	−0.10	−0.25	−0.21	−0.25	0.11	0.10
Agility points	0.07	0.01	0.18	0.22	0.06	−0.11	0.05	−0.10	−0.18	0.01

* r—Pearson’s linear correlation coefficient, *p* < 0.05.

**Table 4:** Pearson’s r linear correlations between performance test results and standardized (Z-score) self-assessment of quality-of-life scores for boys from the study group.

*N* = 64 Boys	W1 Z-Score	W2 Z-Score	W3 Z-Score	W4 Z-Score	W5 Z-Score	W6 Z-Score	W7 Z-Score	W8 Z-Score	W9 Z-Score	W10 Z-Score
Balance (points-pts)	−0.01	−0.06	0.04	−0.12	−0.14	−0.13	0.12	0.07	0.08	−0.06
Upper Limb Movement Speed (pts)	0.01	−0.07	−0.08	0.05	−0.19	0.03	0.10	0.09	0.05	0.00
Flexibility (pts)	−0.01	0.18	0.18	0.10	0.16	0.00	0.00	0.01	0.20	0.09
Jumping Ability (pts)	0.05	−0.05	0.13	0.11	−0.12	−0.14	0.04	0.02	−0.01	−0.10
Hand Strength (pts)	0.13	0.22	0.28 *	0.23	0.11	0.34 *	0.11	0.20	0.26 *	0.10
Trunk Strength (pts)	0.05	0.08	0.33 *	0.24	−0.01	0.05	0.05	0.14	0.13	0.00
Functional Strength (pts)	−0.01	0.05	0.10	0.22	0.08	−0.02	−0.15	0.03	−0.07	0.02
Agility points	0.04	−0.07	0.04	−0.01	−0.10	−0.10	0.18	0.14	0.04	−0.04

* r—Pearson’s linear correlation coefficient, *p* < 0.05.

**Table 5:** Pearson’s r linear correlations between performance test results and standardized (Z-score) HRQOL scores as assessed by parents of girls from the study group.

*N* = 59 Parents of Girls	W1 Z-Score	W2 Z-Score	W3 Z-Score	W4 Z-Score	W5 Z-Score	W6 Z-Score	W7 Z-Score	W8 Z-Score	W9 Z-Score	W10 Z-Score
Balance (points-pts)	0.11	−0.02	0.09	−0.25	0.00	−0.04	−0.04	−0.04	0.04	−0.01
Upper Limb Movement Speed (pts)	−0.11	−0.04	0.05	0.13	0.07	−0.05	0.01	0.00	0.02	0.04
Flexibility (pts)	0.11	0.15	0.35 *	0.32 *	0.12	−0.10	0.10	0.06	0.23	0.04
Jumping Ability (pts)	0.04	−0.03	0.07	0.00	0.12	−0.23	0.00	−0.12	−0.12	−0.16
Hand Strength (pts)	0.01	−0.01	0.02	0.12	0.27 *	0.04	0.20	0.29 *	0.04	0.16
Trunk Strength (pts)	−0.06	−0.03	0.08	0.04	−0.01	−0.26 *	−0.05	−0.07	−0.31 *	0.04
Functional Strength (pts)	−0.06	−0.25	−0.20	−0.03	−0.02	−0.26 *	−0.12	−0.08	−0.09	−0.02
Agility (points)	0.05	0.04	0.11	−0.05	−0.06	−0.23	−0.05	−0.24	−0.28 *	−0.22

* r—Pearson’s linear correlation coefficient, *p* < 0.05.

**Table 6:** Pearson’s r linear correlations between the results of the performance test and the standardized (Z-score) HRQOL results as assessed by parents of boys from the study group.

*N* = 64 Parents of Boys	W1 Z-Score	W2 Z-Score	W3 Z-Score	W4 Z-Score	W5 Z-Score	W6 Z-Score	W7 Z-Score	W8 Z-Score	W9 Z-Score	W10 Z-Score
Balance (points pts)	0.13	−0.04	0.00	0.03	0.03	−0.17	0.07	0.18	−0.04	−0.01
Upper Limb Movement Speed (pts)	0.02	−0.02	−0.01	0.05	−0.13	0.10	−0.07	0.12	0.09	0.06
Flexibility (pts)	0.09	0.16	0.27 *	0.26 *	0.01	0.09	−0.19	0.03	0.03	0.18
Jumping Ability (pts)	−0.02	−0.04	0.10	0.18	0.00	−0.09	−0.17	0.04	−0.07	0.08
Hand Strength (pts)	0.08	0.25 *	0.27 *	0.25 *	0.02	0.27 *	−0.02	0.21	0.17	0.23
Trunk Strength (pts)	0.12	0.08	0.24	0.33 *	0.04	0.07	−0.16	0.08	0.04	0.16
Functional Strength (pts)	0.15	0.01	0.12	0.16	0.12	0.05	−0.12	0.06	0.02	−0.06
Agility (points)	−0.01	−0.08	0.01	0.05	−0.02	−0.07	−0.04	0.11	0.02	−0.02

* r—Pearson’s linear correlation coefficient, *p* < 0.05.

## Data Availability

The original contributions presented in this study are included in the article. Further inquiries can be directed to the corresponding author.

## Abbreviations

The following abbreviations are used in this manuscript:

HRQoL	Health-related quality of life
W1	Physical well-being
W2	Psychological well-being
W3	Moods and emotions
W4	Self-perception
W5	Autonomy
W6	Parent relations and home life
W7	Social support and peers
W8	School environment
W9	Social acceptance (including bullying)
W10	Financial resources
BMI	Body mass index
AWF	Academy of Physical Education
HR-F	Health Related Fitness
IDF	International Diabetes Federation
IPCZD	The Children’s Memorial Health Institute
KBE	The local Bioethical Committee
SD	Standard Deviation
TBW	Total Body Water
WC	Waist Circumference
WHO	World Health Organization
WHR	Waist-to-Hip Ratio
WHtR	Waist-to-Height Ratio/Waist-circumference-to-body-height ratio
